# A concise method for fully automated radiosyntheses of [^18^F]JNJ-46356479 and [^18^F]FITM *via* Cu-mediated ^18^F-fluorination of organoboranes†

**DOI:** 10.1039/d0ra04943c

**Published:** 2020-07-02

**Authors:** Gengyang Yuan, Timothy M. Shoup, Sung-Hyun Moon, Anna-Liisa Brownell

**Affiliations:** Gordon Center for Medical Imaging, Massachusetts General Hospital and Harvard Medical School 3^rd^ Avenue Charlestown MA 02129 USA gyyuan@mgh.harvard.edu abrownell@mgh.harvard.edu

## Abstract

A modified alcohol-enhanced ^18^F-fluorodeboronation has been developed for the radiosyntheses of [^18^F]JNJ-46356479 and [^18^F]FITM. Unlike the [^18^F]KF/K_222_ approach, this method tolerates the presence of sensitive heterocycles in Bpin precursors 4 and 8 allowing a one-step ^18^F-fluorodeboronation on the fully automated TRACERlab™ FX_FN_ platform.

## Abstract 2

A modified alcohol-enhanced ^18^F-fluorodeboronation has been developed for the radiosyntheses of [^18^F]JNJ-46356479 and [^18^F]FITM.

## Graphical abstract

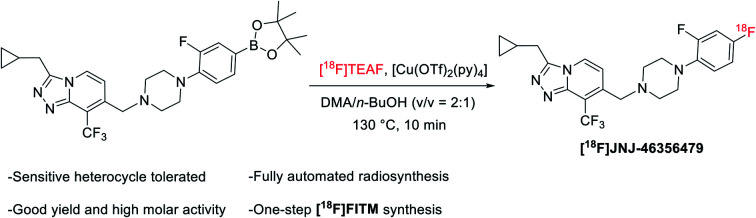

## Introduction

**Fig. 1:**
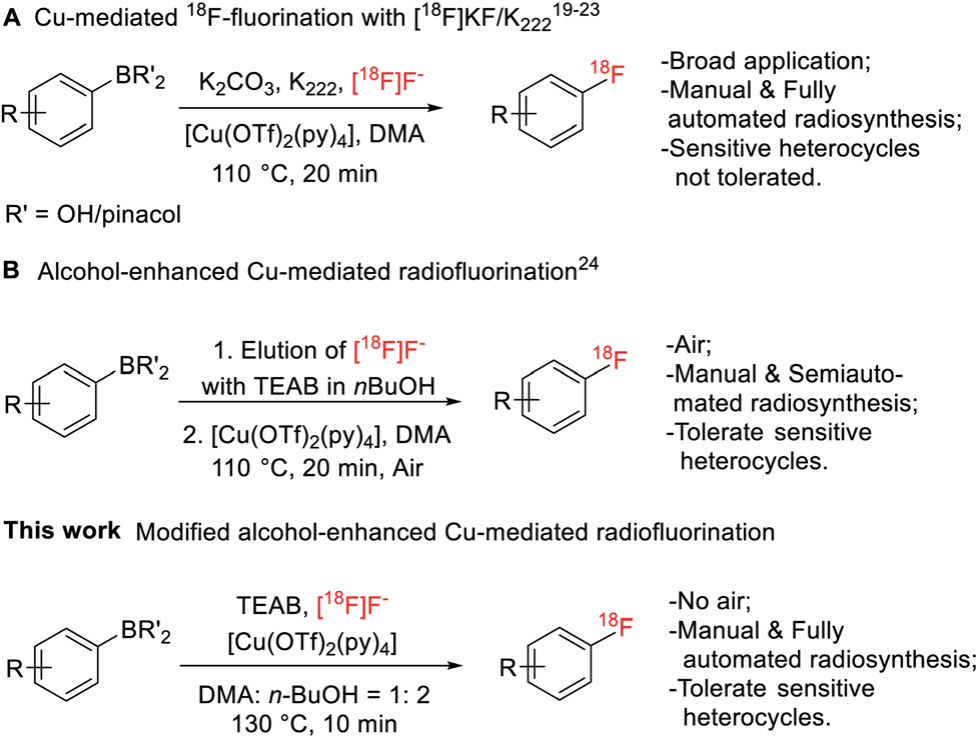
Cu-mediated ^18^F-fluorination of organoboranes.

Fluorine-containing functionalities, especially (hetero)aryl fluorides, are widely present among important drugs and bioactive molecules.^1^ In medicinal chemistry, fluorine is introduced into a compound to enhance chemical and biological properties, such as activity, metabolism stability and bioavailability.^2,3^ Fluorine atom substitution by ^18^F enables the use of positron emission tomography (PET) to image biological processes at the molecular level.^4–8^ Radiolabeling of (hetero)aryl fluorides has improved significantly, allowing incorporation of ^18^F on precursors of aryliodonium salts,^9^ aryliodonium ylides,^10^ aryl-Pd/Ni complexes,^11,12^ triarylsulfonium salts/diaryl sufoxides,^13,14^ and aryl boronic acid/ester/tin species.^15^ Among these methods, the Cu-mediated ^18^F-fluorination of organoboranes has been used for the preparation of various radiopharmaceuticals under manual and automated settings,^16–18^ including eight clinically relevant radioligands^19^ and the recent publications on [^18^F]TRACK,^20^ a tropomyosin receptor kinase inhibitor, and [^18^F]olaparib,^21^ the poly(ADP-ribose) polymerase inhibitor (Fig. 1A). However, in cases of challenging heteroarenes, optimal retro-radiosynthetic routes^22^ and alternative radiolabeling strategies^23^ must be implemented to maximize radiofluorination. For example, the radiolabeling of 4-[^18^F]fluoro-*N*-[4-[6-(isopropylamino)pyrimidin-4-yl]-1,3-thiazol-2-yl]-*N*-methylbenzamide ([^18^F]FITM) and [^18^F]Risperidone with this method is carried out in a two-step manner by first radiolabeling the heterocycle containing fragments, followed by a subsequent coupling step with their corresponding counterparts.^23^

Application of Cu-mediated ^18^F-fluorination in automated platforms is essential in producing large-scale radioligands to support preclinical and clinical studies. Successful automated radiosyntheses with this method are the preparation of [^18^F]TRACK on TRACERlab™ FX_FN_ platform^20^ and the synthesis of [^18^F]olaparib on Ziegler Modular-Lab.^21^ In these reports, automated S_N_Ar radiofluorination occurred with the [^18^F]KF/Kryptofix® 222 (K_222_) complex. Recently, Zischler *et al.* revealed that the milder [^18^F]TEAF complex was also compatible with this radiolabeling method (Fig. 1B).^24^ Moreover, addition of alcohols, such as methanol and *n*-butanol, as co-solvents, seemed to enhance the functionality tolerance of this ^18^F-fluorodeboronation method to include indoles, phenols, and anilines radiolabeled *via* unprotected precursors. However, the authors investigated this approach manually where extensive physical operations were required. For example, the aqueous ^18^F was loaded onto the quaternary methyl ammonium (QMA) cartridge, an anion-exchanger to enrich ^18^F from [^18^O]H_2_O, from the male side instead of the female side followed by an alcohol wash and air flush from the female side to remove the [^18^O]water and increase the recover yield of ^18^F. Air was also introduced into the reactor to promote reaction. These special and tedious operations hinder its application toward the fully automated synthetic modules such as GE TRACERlab™ FX_FN_ platform. Nevertheless, this strategy has been recently utilized by Bernard-Gauthier *et al.* for the radiosynthesis of [^18^F]TRACK using semiautomated radiosynthesis module Scintomics GRP (Germany).^20^

To investigate the suitability of [^18^F]JNJ-46356479 as a PET imaging ligand for metabotropic glutamate receptor 2 (mGluR2) in the brain, we modified this alcohol-enhanced Cu-mediated ^18^F-fluorination to (a) utilize its potential in tolerating sensitive organoboranes that are not compatible with the [^18^F]KF/K_222_ system, and (b) avoid manual manipulations to allow a fully automated radiosynthesis on a GE TRACERlab™ FX_FN_ platform (Fig. 1). After a thorough investigation of radiofluorination conditions for [^18^F]JNJ-46356479, a fully automated method was developed, which was also applicable to the automated one-step radiosynthesis of a mGluR1 negative allosteric modulator (NAM) [^18^F]FITM.

## Results

### Chemistry

JNJ-46356479 was reported by Cid *et al.* as a potent mGluR2 positive allosteric modulator (PAM) (*i.e.*, EC_50_ = 78 nM, *E*_max_ = 256%) with favorable physiochemical and pharmacological properties as well as CNS-penetrant.^25^ JNJ-46356479 has also been established as a selective blocking reagent to characterize the mGluR2 radioligand of [^11^C]JNJ42491293 in rats and non-human primates.^26^ Hence, ^18^F-radiolabeled JNJ-46356479 is an attractive mGluR2 PET radiotracer if produced in high molar activity using a fully automated platform.

**Scheme 1:**
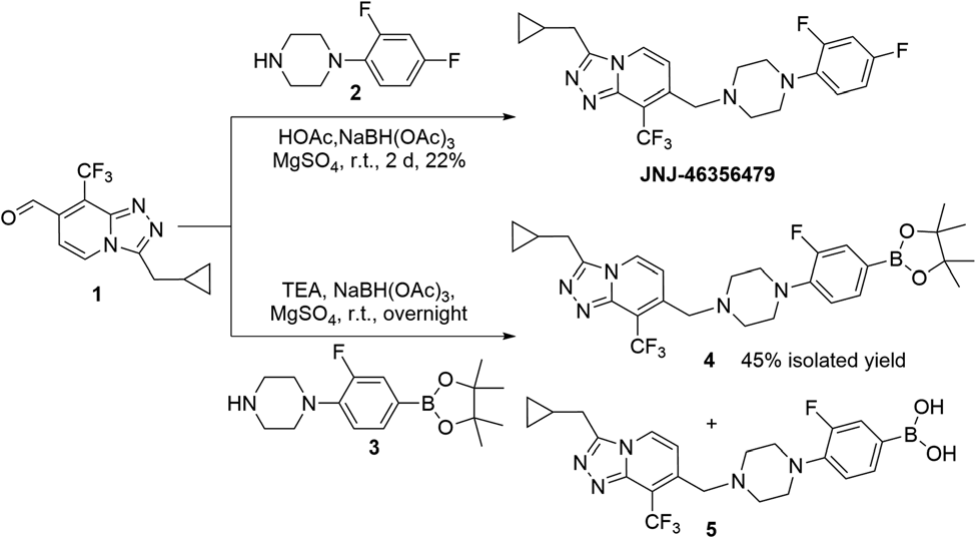
Synthesis of JNJ-46356479 and its boronic pinacol ester precursor 4.

The synthesis of non-labeled JNJ-46356479 was achieved *via* the method reported by Cid *et al.*, starting from 2,4-dichloro-3-(trifluoromethyl) pyridine using over 6 steps to allow final reductive coupling reaction between aldehyde 1 and 1-(2,4-difluorophenyl) piperazine 2 (Scheme 1).^25^ The *para*-aryl fluoride of JNJ-46356479 was selected as a radiolabeling site to avoid steric hinderance when introducing ^18^F or bulky leaving groups. Traditional nucleophilic S_N_Ar substitution of nitro- or iodo-leaving groups was assumed not to be feasible due to the poor activating effect of adjacent fluoride. The sulfonium or iodonium salt or iodonium ylide radiolabeling methods, however, required oxidative reaction conditions that might not be tolerated by the heterocycle containing JNJ-46356479.

**Scheme 2:**
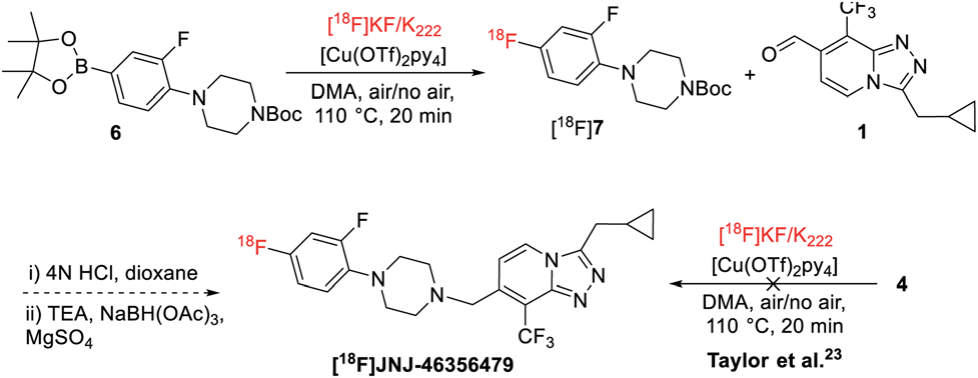
Radiofluorination of [^18^F]JNJ-46356479.

The ^18^F-fluorodeboronation precursor 4 was synthesized in a similar manner as that of JNJ-46356479 with the boronic ester 3. However, direct application of the same reductive amination reaction conditions, using HOAc and NaBH(OAc)_3_, resulted in substantial side products that were in close vicinity with the desired product 4, including the deboronation product (aryl-H) and boronic species 5. Interestingly, this decomposition did not occur to the structurally simpler fragment 3, which was prepared *via* the de-Boc protection of compound 6 under strong acidic condition at room temperature (Scheme 2). To improve the yield of compound 4, the basic reductive amination conditions with triethylamine (TEA) and NaBH(OAc)_3_ were applied, resulting in much less side products and an increased yield of compound 4.

### Radiosynthesis of [^18^F]JNJ-46356479

Initial radiofluorination of compound 4 with this ^18^F-fluorodeboronation using K_2_CO_3_ as a base and K_222_ as a phase transfer reagent, however, failed to give the desired [^18^F]JNJ-46356479 under the literature conditions (Scheme 2).^19,23^ As suggested by Taylor *et al.*, heterocyclic complex molecules might be sensitive, therefore, not tolerated by direct ^18^F-fluorodeboronation with [^18^F]KF/K_222_.^23^ In this case, a multistep radiolabeling protocol is required by radiolabeling the heterocycle 6 first, followed by *N*-deprotection and *N*-alkylation coupling with aldehyde 1. Indeed, under the same conditions, fragment 6 could be radiolabeled to give [^18^F]7 with a radiochemical conversion (RCC) of 60 ± 5% (*n* = 2). It is therefore reasonable to assume the [1,2,4]triazolo[4,3-*a*]pyridine core structure of JNJ-46356479 is vulnerable under the suggested [^18^F]KF/K_222_ radiofluorination conditions. Before resorting to the complicated and tedious two-step strategy, we intended to explore the alcohol-enhanced protocol reported by Zischler and coworkers^24^ to find solutions for the one-step radiosynthesis of [^18^F]JNJ-46356479.

The original manual radiolabeling protocol was not directly applied for [^18^F]JNJ-46356479 due to the reproducibility issue witnessed during the radiofluorination of compound 6 and incomplete removal of [^18^O]H_2_O from QMA cartridge by air would inhibit the reaction. Instead, the labeling protocol was comprehensively investigated to reveal critical influential factors that would affect the reaction outcomes. More importantly, efforts were focused on simplifying the manual handlings of this method to apply it to the fully automated synthetic modules.

As shown in Table 1, based on the original reaction conditions, the reaction temperature, time as well as the amounts of TEAB, precursor 4 and catalyst [Cu(OTf)_2_py_4_] were optimized. The anhydrous dimethylacetamide (DMA) was used as a solvent. The optimal reaction temperature was 130 °C and further elevation of reaction temperature did not significantly increase the RCCs (entries 1–3). Increasing the amount of base was detrimental to the RCCs (entry 4), though it could improve the elution efficiency of ^18^F from QMA cartridge (∼95–99%). On the other hand, increased precursor amount was beneficial to enhance the RCCs (entries 5–8). These results indicated that boronic pinacol ester (Bpin) 4 was vulnerable to the excess amount of base. This sensitivity of organoboranes was also noted by Zhang *et al.*, where use of Py-OTf or DMAP-OTf as ^18^F eluting agent and base alleviated this issue.^27^ To maintain a balanced recovery of ^18^F (85–90%) and to limit precursor use, 3.5 mg (6.25 μmol) of 4 and 2.7 mg (14.1 μmol) of TEAB were considered as optimal amounts (entry 8). The reaction time showed little effect on the resulting RCCs (entries 9–11), thus a shorter reaction time was preferred. The amount of [Cu(OTf)_2_(py)_4_] was also optimized, where reduction of the catalyst by half (*i.e.*, 9 mg, 13.3 μmol) led to similar RCCs (entries 12–14). In addition, the boronic acid precursor 5 was also examined for the synthesis of [^18^F]JNJ-46356479 with the same method. Although MeOH was claimed as an accompanying alcohol for boronic acid precursors by Zischler and coworkers,^24^ it failed to give the desired product in this radiofluorination reaction (Table 1, entry 15; Table S2,† entries 1 and 2). However, *n*-BuOH led to the formation of [^18^F]JNJ-46356479 (Table 1, entry 16; Table S2,† entries 3–6). The RCCs ranged from 5% to 13% with elevated temperature and increased amount of precursor favored the radiochemical conversion (Table S2,† entries 3–6). Therefore, it was reasonable to conclude that the boronic pinacol ester 4 was more reactive than the boronic acid 5 for this radiofluorination method.

**Table 1:** Optimization of the one-step Cu-mediated ^18^F-fluorination of JNJ-46356479 with organoboranes

Entry	Precursor (mg per equiv.)	TEAB (mg per equiv.)	[Cu(OTf)_2_(py)_4_] (mg per equiv.)	Co-solvent (mL)	*T* (°C)	*t* (min)	RCC by rTLC (%)
1	4 (3.0/1.0)	2.7/2.6	18.0/4.9	*n*-BuOH (0.4)	110	20	22 ± 2 (*n* = 2)
2	4 (3.0/1.0)	2.7/2.6	18.0/4.9	*n*-BuOH (0.4)	130	20	25 ± 2 (*n* = 5)
3	4 (3.0/1.0)	2.7/2.6	18.0/4.9	*n*-BuOH (0.4)	150	20	20 ± 2 (*n* = 2)
4	4 (3.0/1.0)	8.2/7.8	18.0/4.9	*n*-BuOH (0.4)	130	20	7 ± 2 (*n* = 2)
5	4 (0.5/1.0)	2.7/15.6	18.0/29.4	*n*-BuOH (0.4)	130	20	2 ± 1 (*n* = 2)
6	4 (1.0/1.0)	2.7/7.8	18.0/14.7	*n*-BuOH (0.4)	130	20	5 ± 1 (*n* = 2)
7	4 (1.5/1.0)	2.7/5.2	18.0/9.8	*n*-BuOH (0.4)	130	20	12 ± 1 (*n* = 2)
8	4 (3.5/1.0)	2.7/2.3	18.0/4.3	*n*-BuOH (0.4)	130	5	28 ± 3 (*n* = 2)
9	4 (2.0/1.0)	2.7/3.9	18.0/7.4	*n*-BuOH (0.4)	130	5	15 ± 1 (*n* = 2)
10	4 (2.0/1.0)	2.7/3.9	18.0/7.4	*n*-BuOH (0.4)	130	10	14 ± 2 (*n* = 2)
11	4 (2.0/1.0)	2.7/3.9	18.0/7.4	*n*-BuOH (0.4)	130	20	15 ± 1 (*n* = 2)
12	4 (2.0/1.0)	2.7/3.9	4.5/1.8	*n*-BuOH (0.4)	130	20	6 ± 1 (*n* = 2)
13	4 (2.0/1.0)	2.7/3.9	9.0/3.6	*n*-BuOH (0.4)	130	20	21 ± 1 (*n* = 2)
14	4 (3.5/1.0)	2.7/2.3	9.0/2.1	*n*-BuOH (0.4)	130	10	28 ± 2 (*n* = 4)
15	5 (2.0/1.0)	2.7/3.4	18.0/6.3	MeOH (0.4)	110	10	0 (*n* = 2)
16	5 (5.0/1.0)	2.7/1.3	18.0/2.5	*n*-BuOH (0.4)	150	20	13 ± 2 (*n* = 3)

Noteworthy, the ^18^F in [^18^O]water was loaded onto the QMA cartridge from the female side and eluted out from the male side in the normal manner. The [^18^F]TEAF complex was obtained after conventional azeotropic dryings and no air was flushed into the reactor during reaction. These modifications facilitated the subsequent utilization of fully automated synthetic modules.

Automated radiosynthesis of [^18^F]JNJ-46356479 was performed with the optimal conditions depicted in Table 1 entry 14, using a computer controlled GE TRACERlab™ FX_FN_ module (see ESI, Fig. S1†). The final [^18^F]JNJ-46356479 was obtained with a radiochemical yield (RCY) of 5 ± 3% (*n* > 10, non-decay-corrected), a molar activity of 180 ± 102 GBq μmol^−1^ at end of synthesis (EOS, 45 min, *n* > 10) and excellent chemical and radiochemical purities (>95%, see ESI, Fig. S3†). Noteworthy, it was crucial to add *n*-BuOH first to dissolve the azeotropically dried [^18^F]TEAF complex before applying the precursor solution, otherwise only negligible amount of [^18^F]JNJ-46356479 (RCY < 0.1%, *n* = 2) would be obtained (see ESI, Fig. S4†).

### Radiosynthesis of [^18^F]FITM

**Scheme 3:**
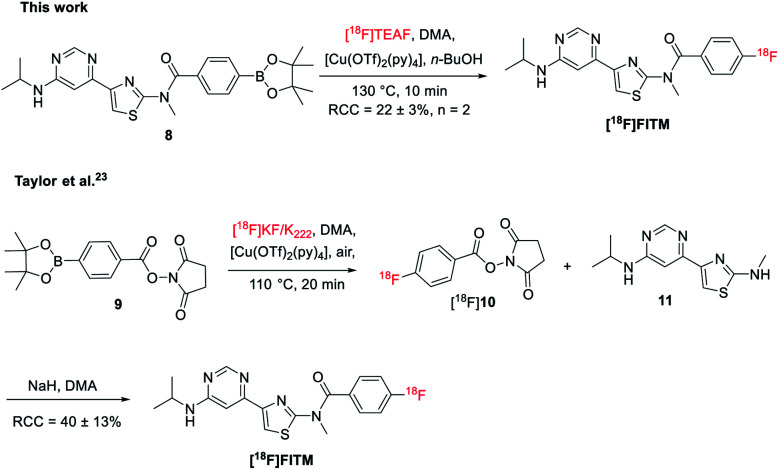
Cu-mediated ^18^F-fluorination of [^18^F]FTIM.

To further test the applicability of this method to sensitive heterocycle containing molecules, the automated radiosynthesis of [^18^F]FITM was also investigated. The Bpin precursor 8 was synthesized in 3 steps from the 4-(6-chloropyrimidin-4-yl)-*N*-methylthiazol-2-amine *via* the literature method (Scheme 3).^23^ As reported by the Taylor *et al.*, direct radiofluorination of 8 with the [^18^F]KF/K_222_ system failed to give the desired product.^23^ Alternatively, [^18^F]FTIM was prepared by a three-step one-pot manner, where the benzoate fragment 9 was first radiolabeled by [^18^F]KF/K_222_ to give [^18^F]10, followed by acidification and amidation with the intermediate 11 under basic conditions (Scheme 3). The final [^18^F]FTIM was afforded with a RCC of 40 ± 13% over the three-step synthesis. On the other hand, when the optimized reaction conditions as those of [^18^F]JNJ-46356479 were applied (Table 1, entry 14), [^18^F]FTIM was synthesized in one-step from 8 with a RCC of 22 ± 3% (Scheme 3, *n* = 2, non-decay-corrected). The radiosynthesis was carried out in the automated TRACERlab™ FX_FN_ module in a similar manner as that of [^18^F]JNJ-46356479 (see ESI, Fig. S5†). The successful synthesis of [^18^F]FTIM further demonstrated this modified alcohol-enhanced Cu-mediated ^18^F-fluorination could allow the radiofluorination of a broader organoboranes substrates.

## Discussion

The alcohol enhanced Cu-mediated radiofluorination reported by Zischler and coworkers has been used for the radiosynthesis of 6-[^18^F]FDOPA^24^ manually with its Bpin precursor. This Bpin precursor also tolerated the [^18^F]KF/K_222_ involving conditions^15,19^ as well as [^18^F]TEAF nucleophile derived from a mixture of TEAOTf and Cs_2_CO_3_ in DMF/pyridine solution,^28^ where fully automated radiosynthesis could be achieved. Likewise, the aforementioned [^18^F]TRACK^20^ could also be obtained from its Bpin precursor with [^18^F]KF/K_222_ nucleophile under either manual or automated labeling settings. Distinct from these examples, the radiofluorination of the Bpin precursors for JNJ-46356479 and FITM were not tolerated by the broadly used [^18^F]KF/K_222_ nucleophiles. We assumed the sensitive heterocycle moieties in the precursors 4 and 8 attributed to the unsuccessful ^18^F-fluorodeboronation *via* [^18^F]KF/K_222_. Under such conditions, the Bpin group decomposed immediately from its parent precursor before ^18^F was incorporated.

In fact, the presence of sensitive heterocycles also made compounds 4 and 8 chemically unstable under certain conditions. They slowly decomposed to their aryl boronic acid analogues during flash column purification with either silica gel or alumina. Initial purifications of 4*via* a preparative C-18 column with a gradient elution (mobile phase: CH_3_CN/0.1% formic acid in water, flow rate: 15 min mL^−1^) resulted in a 75% degradation of 4 to its boronic acid analog 5 after the collected product fractions were dried in lyophilizer overnight. Although the boronic pinacol esters are generally considered more stable than boronic acids under protodeboronation conditions,^29^ the protodeboronation side product was minor (<5%). In addition, slight decompositions were also noticed for 4 and 8 during NMR characterizations in deuterated solvents of CDCl_3_, CD_3_OD and dimethyl sulfoxide-d_6_. In practice, the Bpin precursors were purified *via* flash column chromatography immediately after workup and characterized by LC-MS and NMR for their purities and identities. Fortunately, the boronic acid analogues were also suitable for the radiofluorination method as tested here with compound 5. Moreover, once isolated, both precursors are quite stable at room temperature. During the reaction, compounds 4 and 8 maintained a high purity of >95%, with marginal amount of their boronic acid analogues. Although pure compound 5 was not directly tested under the [^18^F]KF/K_222_ conditions, a mixture containing 62% of 4, 35% of 5 and 3% of their protodeboronation side product did not lead to the desired [^18^F]JNJ-46356479, whereas under the optimized reaction conditions shown in Table 1 entry 14, [^18^F]JNJ-46356479 can be obtained with the same mixture.

Switching the base from K_2_CO_3_ to TEAB together with the addition of *n*-BuOH had alleviated the harshness of the reaction conditions and led to the desired products from their Bpin precursors. Even under this modified protocol, the amount of TEAB had to be limited to ensure an optimal RCC (Table 1, entry 4). The *n*-BuOH was positioned separately in vial 2 and added to reaction vessel before the precursor solution in the FX_FN_ platform, which proved to be crucial, otherwise only negligible [^18^F]JNJ-46356479 would be obtained. The successful automated radiosynthesis of [^18^F]JNJ-46356479 and [^18^F]FTIM demonstrated that employment of traditional ^18^F trap & release on QMA cartridge, conventional azeotropic drying, as well as devoid of air flush to the reaction mixture, was applicable to the alcohol-enhanced ^18^F-fluorodeboronation.

## Conclusions

The modified alcohol-enhanced Cu-mediated ^18^F-fluorination here is succinct, robust, and highly producible. It allows the large-scale production of [^18^F]JNJ-46356479 in a range of 1.1 GBq to 3.0 GBq from a single batch with sufficiently high molar activity (180 ± 102 GBq μmol^−1^) to satisfy preclinical and clinical usage. Considering the important role of mGluR2 in various psychiatric and neurological disorders^30–33^ and the lack of efficient mGluR2 PET imaging ligands to visualize and quantify mGluR2 in the brain,^26^ the impact of this work is substantial. Moreover, the successful one-step radiosynthesis of [^18^F]FTIM by this method demonstrates its broader organoborane compatibility. Along with this work, the preclinical characterization of [^18^F]JNJ-46356479 as a mGluR2 PET imaging ligand in rodents and monkey brains has been performed and will be disclosed in due course.

## Conflicts of interest

There are no conflicts to declare.

## Supplementary Material

RA-010-D0RA04943C-s001
