# Supplementary material for: A concise method for fully automated radiosyntheses of [18F]JNJ-46356479 and [18F]FITM via Cu-mediated 18F-fluorination of organoboranes
Source: RSC Adv. 2020 Jul 2;10(42):25223–7. doi: 10.1039/d0ra04943c (PMC7497470; doi:10.1039/d0ra04943c)
Supplement: RA-010-D0RA04943C-s001 [file RA-010-D0RA04943C-s001.pdf]

Electronic Supplementary Material (ESI) for RSC Advances.
This journal is © The Royal Society of Chemistry 2020

A concise method for fully automated radiosyntheses of [^{18}F]JNJ-46356479 and [^{18}F]FITM via Cu-mediated ^{18}F-fluorination of organoboranes

Gengyang Yuan,* Timothy M. Shoup, Sung-Hyun Moon, Anna-Liisa Brownell*

Gordon Center for Medical Imaging, Massachusetts General Hospital and Harvard Medical School, 3rd Avenue, Charlestown, MA 02129, USA

Corresponding Authors

*G.Y.: phone, 857-210-6386; e-mail, gyyuan@mgh.harvard.edu

*A-L.B.: phone, 617-744-3725; e-mail, abrownell@mgh.harvard.edu

Table of Contents

General Information……………………………………………………………………….3

Organic Chemistry……………………………………………………………...……….4

Radiochemistry…………………………………………………………………………6

[18F]fluoride in [18O]H_2O…………………………………………………………...6

Manual radiosynthesis of [18F]JNJ-46356479 from Bpin **4**……………………………………6

Automated radiosynthesis of [18F]JNJ-46356479 from Bpin **4**………………..…………………6

The effect of the order of adding *n*BuOH……………………………...……………10

Manual radiosynthesis of [18F]JNJ-46356479 from boronic acid **4**…………..………………..11

Automated Radiosynthesis of [18F]FITM ……………………………………………11

Optimization of the radiosynthesis of [18F]JNJ-46356479 with **4**………………………………12

Optimization of the radiosynthesis of [18F]JNJ-46356479 with **5**………………………………14

References…………………………………………………………………………….15

NMR spectra…………………………………………………………..………..……16

GENERAL INFORMATION

All reagents and starting materials were obtained from the commercial sources including Sigma-Aldrich (St. Louis, MO), Thermo Fisher Scientific, Oakwood Products, Inc., Matrix Scientific, Acros Organics and used as received. The standard FITM was purchased from Tocris Bioscience. The reactions were monitored by TLC using a UV lamp monitored at 254 nm. If necessary, the reactions were also checked by LC-MS using the Agilent 1200 series HPLC system coupled with a multi-wavelength UV detector and a model 6310 ion trap mass spectrometer (Santa Clara, CA) equipped with an Agilent Eclipse C8 analytical column (4.6 × 150 mm, 5 μm). Elution was with a 0.1% formic acid solution of water (A) and acetonitrile (B). The radioTLC experiments were performed on EMD TLC Silica gel 60 plates using a Bioscan AR-2000 radio-TLC imaging scanner and WinScan software. The identity and purity of [^{18}F]JNJ-46356479 and [^{18}F]FITM were determined by ThermoFisher UltiMate 3000 HPLC system together with a Bioscan Flow-Count equipped with a NaI crystal, and Breeze software. The silica gel used in flash column chromatography was from Aldrich (Cat. 60737, pore size 60 Å, 230-400 mesh). Flash chromatography was also performed with a CombiFlash R_f Purification System (Teledyne Isco) using a Silica ReadySep R_f column. The products were identified by LC-MS as well as ^{1}H NMR and ^{13}C NMR using either a Bruker 300 MHz spectrometer or a JEOL 500 MHz spectrometer as indicated. All NMR samples were dissolved in chloroform-d ($CDCl_3$), methanol-d$_4$ (CD_3OD) or DMSO-d$_6$ [$(CD_3)_2SO$] containing tetramethylsilane as a reference standard. Chemical shifts were expressed as ppm and calculated downfield or upfield from the NMR signal of reference standard. *J* was expressed as Hz, and its splitting patterns were reported as s, d, t, q, or m. Unless otherwise specified, the purities of all new compounds were over 95% determined by HPLC.

ORGANIC CHEMISTRY

As mentioned above, the syntheses of aldehyde **1**[1] and **8**[2] were achieved following the literature methods. The final products were characterized with LC-MS and ^{1}HNMR, which were consistent as those reported in literature. The rest compounds' characterizations are illustrated below.

1-(2-fluoro-4-(4,4,5,5-tetramethyl-1,3,2-dioxaborolan-2-yl)phenyl)piperazine (3) *tert*-butyl 4-(2-fluoro-4-(4,4,5,5-tetramethyl-1,3,2-dioxaborolan-2-yl)phenyl)piperazine-1-carboxylate (**6**, 0.15 g, 0.38 mmol) was dissolved in 4N HCl in dioxane (8.0 mL). The resulting solution was stirred at room temperature for 1 h. The reaction was monitored via TLC. After the reaction was completed, the dioxane was removed under vacuum to get compound **3** as a HCl salt (0.132g, quantitative). The product was used for the next step without further purification.

3-(cyclopropylmethyl)-7-((4-(2-fluoro-4-(4,4,5,5-tetramethyl-1,3,2-dioxaborolan-2-yl)phenyl)piperazin-1-yl)methyl)-8-(trifluoromethyl)-[1,2,4]triazolo[4,3-a]pyridine (4) To a stirred solution of compound **3** (0.132g, 0.38 mmol) in anhydrous dichloromethane (4.0 mL) under N_2 at room temperature was added triethylamine (0.22 mL, 1.52 mmol), $MgSO_4$ (0.46g, 3.82 mmol) and 3-(cyclopropylmethyl)-8-(trifluoromethyl)-[1,2,4]triazolo[4,3-a]pyridine-7-carbaldehyde (**1**, 0.1g, 0.38 mmol). The reaction mixture was stirred for 30 min, and then $NaBH(OAc)_3$ (0.121g, 0.57 mmol) was added. The reaction mixture was stirred at room temperature overnight and was then diluted with dichloromethane and washed with water and brine and dried with $MgSO_4$. The solvent was removed under vacuum, and the residue was purified by flash chromatography to give the desired product of **4** as a pale-yellow solid (95 mg, 0.17 mmol, 45% yield). ^{1}H NMR (300 MHz, CD_3OD) δ ppm 8.56 (d, J = 7.2 Hz, 1H), 7.52 (d, J = 7.3 Hz, 1H), 7.44 (d, J = 9.2 Hz, 1H), 7.30 (d, J = 13.6 Hz, 1H), 6.87-7.08 (m, 1H), 3.91 (s, 2H), 3.16-3.19 (m, 4H), 3.11 (d, J = 6.9 Hz, 2H), 2.66-2.82 (m, 4H), 1.31 (s, 12H) 1.17-1.31 (m, 1H), 0.59-0.65 (m, 2H), 0.33-0.38 (m, 2H). ^{13}C NMR (125 MHz, CD_3OD) δ ppm 155.9, 153.9, 148.1, 146.2, 142.6, 142.5 (m), 131.2, 125.9, 123.5 (d, J = 240.0 Hz), 121.0 (d, J = 19.1 Hz), 118.0, 115.5, 114.9 (d, J = 34.1 Hz), 83.8, 57.3, 53.0, 49.8, 28.2, 23.9, 7.9, 4.5. LC-MS calculated for $C_{28}H_{34}BF_4N_5O_2$: 559.27; observed: *m/z* 560.1 $[M+H]^+$.

(4-(4-((3-(cyclopropylmethyl)-8-(trifluoromethyl)-[1,2,4]triazolo[4,3-a]pyridin-7-yl)methyl)piperazin-1-yl)-3-fluorophenyl)boronic acid (5) The mixture of compounds **4** and **5** (0.5 g) from lypholizer was purified via silica gel flash chromatography to give **5** as a white solid (0.2 g, 0.42 mmol). ^{1}H NMR (300 MHz, CD_3OD) δ ppm 8.54 (d, J = 7.2 Hz, 1H), 7.53 (d, J = 7.3 Hz, 1H), 7.38 (d, J = 7.7 Hz, 1H), 7.27 (d, J = 13.7 Hz, 1H), 6.95-7.32 (m, 1H), 3.86 (s, 2H), 3.12-3.21 (m, 4H), 3.12 (d, J = 6.9 Hz, 2H), 2.63-2.78 (m, 4H), 1.18-1.39 (m, 3H), 0.57-0.74 (m, 2H), 0.30-0.44 (m, 2H). ^{13}C NMR (75 MHz, CD_3OD) δ ppm 158.0, 154.8, 149.5, 147.5, 144.0 (m), 131.6, 128.5 (d, J = 290.4 Hz), 127.2, 122.9, 122.0 (d, J = 19.0 Hz), 119.3, 116.9, 116.3 (d, J = 33.7 Hz), 58.7, 54.4, 51.5, 29.7, 9.2, 5.4. LC-MS calculated for $C_{28}H_{34}BF_4N_5O_2$: 477.20; observed: *m/z* 478.0 $[M+H]^+$.

RADIOCHEMISTRY

[^{18}F]fluoride in [^{18}O]H_2O

[^{18}F]fluoride was produced by a GE PETtrace 16.5 MeV cyclotron (GE Healthcare, Waukesha, WI, USA) using $^{18}O(p, n)^{18}F$ nuclear reaction to irradiate ^{18}O-enriched water (Isoflex Isotope, San Francisco, CA).

Manual radiosynthesis of [^{18}F]JNJ-46356479 from Bpin 4

[^{18}F]fluoride (370-555 MBq) in [^{18}O]H_2O was trapped on a Waters QMA cartridge and then was released into a V-vial using 1 mL CH_3CN/H_2O (7:3) containing TEAB (2.7 mg, 14.1 μmol). The resulting solution was azeotropically dried at 110 °C with additions of 3 x 1 mL anhydrous CH_3CN in a stream of nitrogen to get the [^{18}F]TEAF complex. 0.4 mL anhydrous *n*-BuOH was then added to this residue, followed by a solution of [$Cu(OTf)_2(py)_4$] (9.0 mg, 13.3 μmol) and **4** (3.5 mg, 6.2 μmol) in 0.8 mL anhydrous dimethylacetamide (DMA). The resulting mixture was stirred at 130 °C for 20 min. The reaction was quenched by addition of 2.3 mL H_2O and analyzed by radioTLC (Figure S1) and analytical radioHPLC to obtain the RCCs and confirm its identity and purity, respectively (mobile phase: 60% CH_3CN in 0.1M AMF water, 1 mL/min, Waters Xbridge BEH C18, 3.5 μm, 4.6 x 150 mm).

Automated radiosynthesis of [^{18}F]JNJ-46356479 from Bpin 4

Automated radiosynthesis of [^{18}F]JNJ-46356479 was performed with the optimal conditions depicted in Table 1 entry 14, using a computer controlled GE TRACERLab^{TM} FX_{FN} module (Figure S1). In practice, the automated module was thoroughly cleaned before each synthetic run. Reagents were then loaded into vials *V1*-*V9*: *V1*: 1 mL of TEAB (2.7 mg) in CH_3CN/water (0.7/0.3), *V2*: 0.4 mL *n*-BuOH, V*3*: 3.5 mg of **4** and 9.0 mg [$Cu(OTf)_2(py)_4$] in 0.8 mL anhydrous *N*,*N*-Dimethylacetamide (DMA) solution, *V4*: 1 mL CH_3CN, *V5*: 3.2 mL water, *V7*: 10 mL water, *V8*: 0.6 mL EtOH, *V9*: 3.0 mL USP saline solution. The round bottom flask *15* and the product vial *17* was pre-filled with 23 mL water and 3.0 mL USP saline, respectively. An activated Waters QMA cartridge was placed at the position of *11*. The activated Waters Alumina N cartridge and

Waters C18 light SepPak cartridge were also put in place (*13* and *16*). A 20 mL sterile empty vial attached with a Millipore Millex® GV membrane filter and a sterile venting needle was connected to the end of the product collecting line at *18*. A semi-preparative HPLC column was connected and equilibrated for 30 min with the mobile phase at a flow rate of 6 mL/min before use.

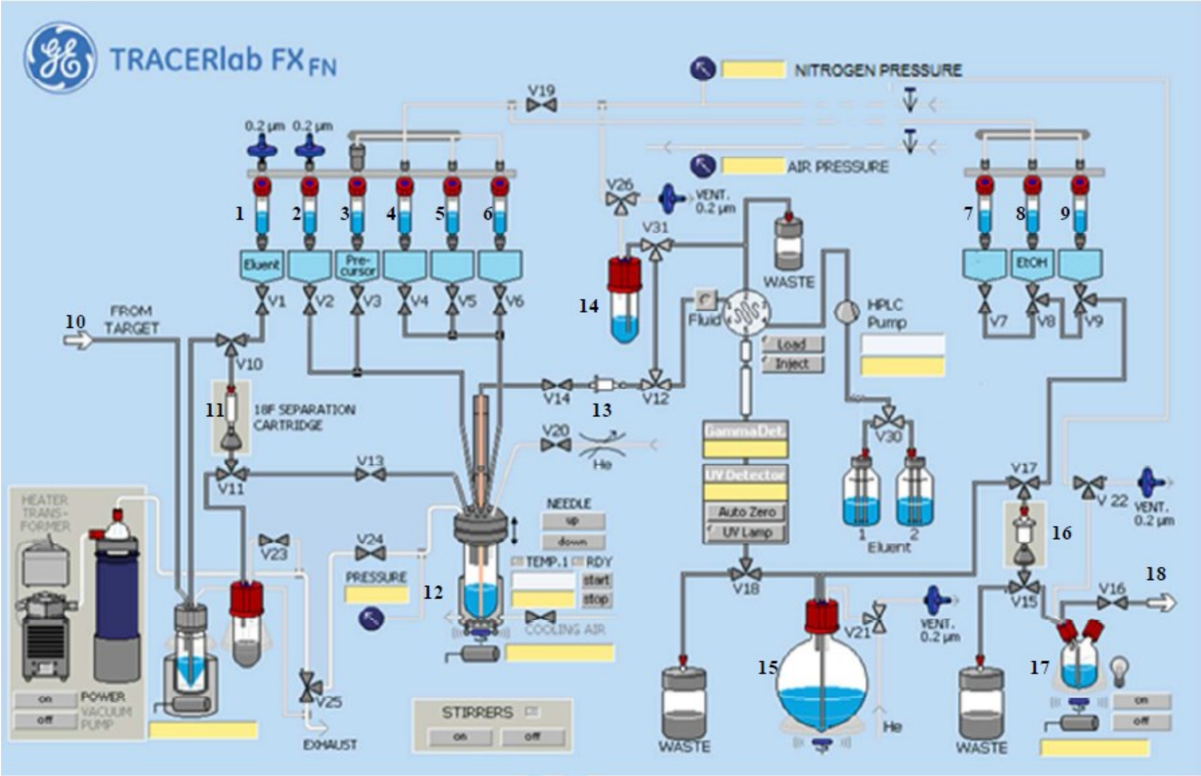

Figure S1. A schematic diagram of the GE medical systems commercial GE TRACERlab™ FX_{FN} radiosynthesis module used for the synthesis of [^{18}F]JNJ-46356479

A bolus of [^{18}O]water containing [^{18}F]fluoride (18.5 GBq ~ 74 GBq) from cyclotron was loaded onto a QMA cartridge and eluted into the graphite reaction vessel with a solution of TEAB in 1 mL CH_3CN/water (7:3). The solvent was azeotropically evaporated under reduced pressure and argon stream. One more portion of CH_3CN (1 mL) was added to the reaction vessel and subsequently evaporated to afford a dry [^{18}F]TEAF residue. After cooling to 50 °C, *n*-BuOH (0.4 mL) was added and stirred for 1 min, followed by a solution of the boronic pinacol ester precursor (**4**, 3.5 mg) and [$Cu(OTf)_2(py)_4$] (9.0 mg) in anhydrous DMA (0.8 mL). The reaction vessel was sealed, and the reaction mixture was stirred and heated at 130 °C for 10 min. After cooling to 50°C,

the mixture was quenched with water (3.2 mL). The reaction mixture was passed through a Waters Alumina N cartridge to remove the unreacted [^{18}F]fluoride. The resulting mixture was then loaded onto a semi-preparative HPLC column (Waters Xbridge BEH C18, 5 µm, 10 x 250 mm) eluting with a mobile phase of 55% CH_3CN in 0.1M AMF water at a flow rate of 6 mL/min. The product fraction (eluting at 10.8 min, Figure S2) was collected and diluted with water (23 mL) in a round bottom flask. The diluted product solution was then passed through a Waters C18 light SepPak cartridge. The SepPak was washed with water (10 mL) and dried. The final product was eluted off the SepPak with EtOH (0.6 mL), followed by USP saline (3.0 mL) into a product vial pre-charged with USP saline (3.0 mL). Finally, the formulated product solution was passed through a membrane filter (Millipore Millex® GV, 0.22 µm) into a vented sterile empty vial.

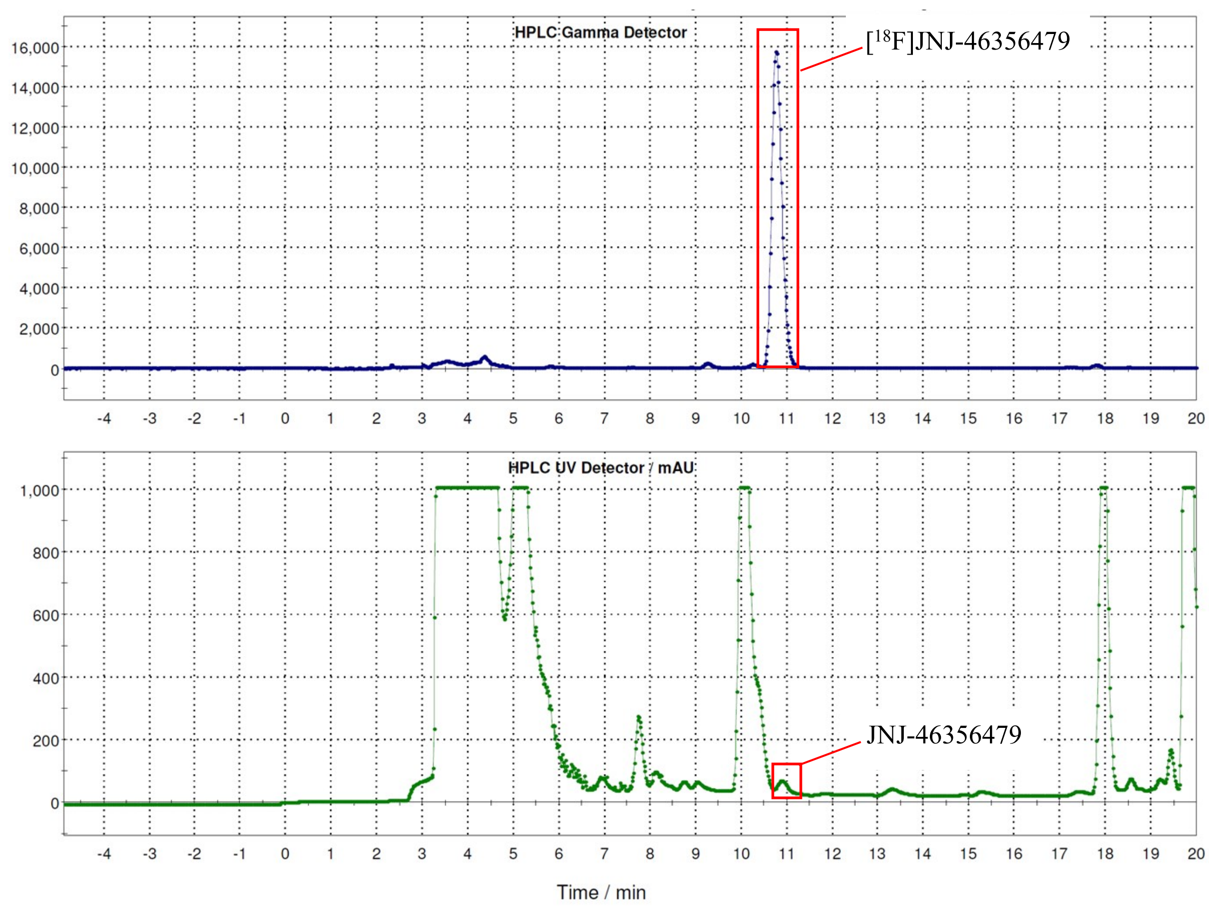

Figure S2. Purification chromatography of [^{18}F]JNJ-46356479 from the reaction mixture via semi-preparative HPLC.

The final [^{18}F]JNJ-46356479 was obtained with a RCY of 5 ± 3% (n > 10, non-decay-corrected), a molar activity of 180 ± 102 GBq/µmol at end of synthesis (EOS, 45 min, n > 10) and excellent chemical and radiochemical purities (> 95%, Figure S3).

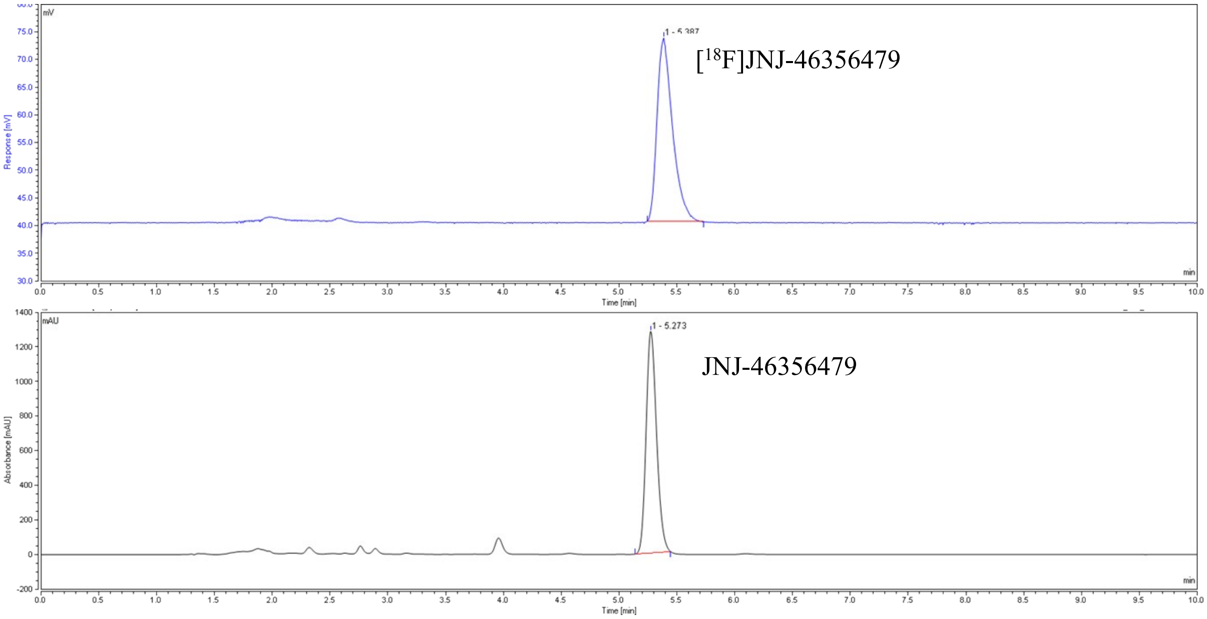

Figure S3. Confirmation of the [^{18}F]JNJ-46356479 via HPLC analysis. The radioHPLC trace is shown in blue (top) and the UV trace is shown in black (bottom) under a wavelength of 254 nM. Mobile phase: 60% CH_3CN in 0.1M AMF water, 1 mL/min, Waters Xbridge BEH C18, 3.5 µm, 4.6 x 150 mm.

The effect of the order of adding *n*BuOH

Noteworthy, it was crucial to add *n*-BuOH first to dissolve the azeotropically dried [^{18}F]TEAF complex before applying the precursor solution, otherwise only negligible amount of [^{18}F]JNJ-46356479 (RCY < 0.1%, n = 2) would be obtained (Figure S4).

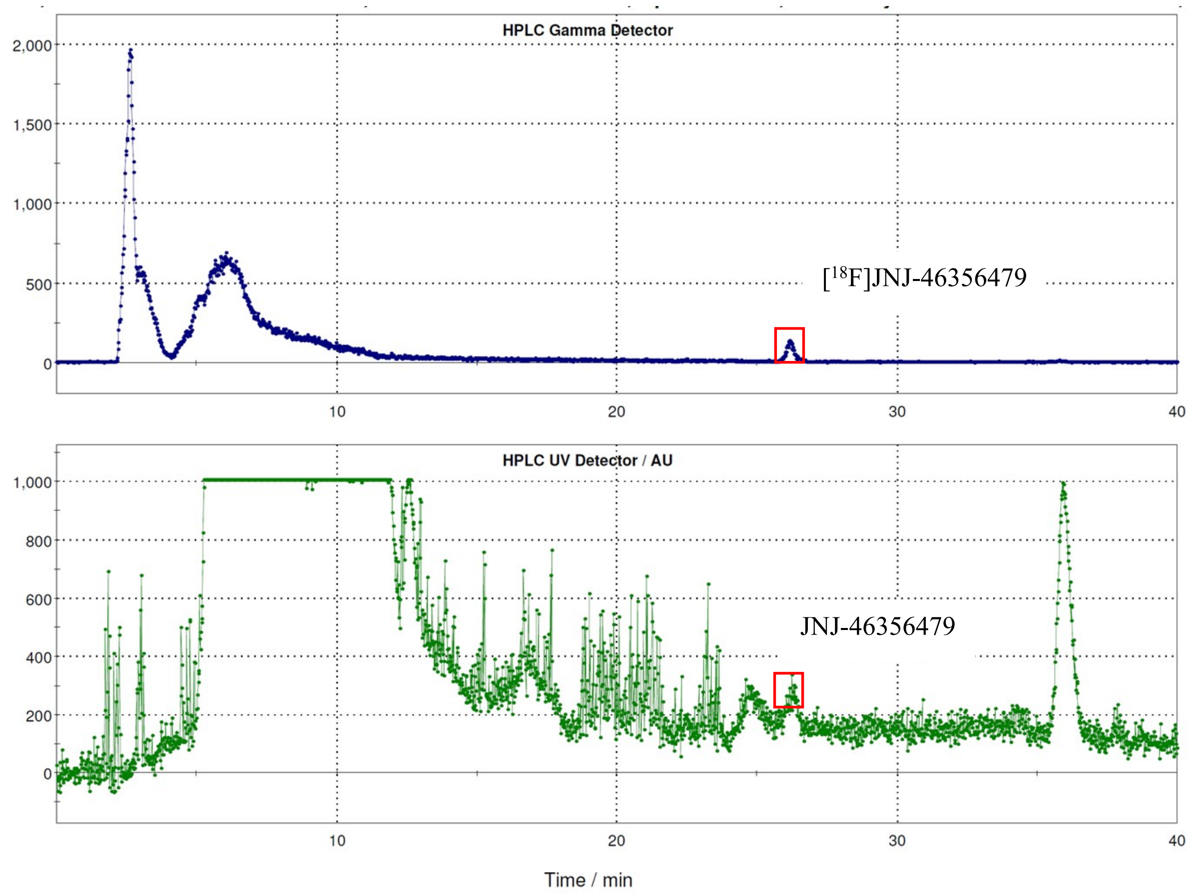

Figure S4. Semi-preparative radioHPLC spectra for the reaction mixture of [^{18}F]JNJ-46356479. Column: Waters Xbridge BEH C18, 5 µm, 10 x 250 mm; Eluent: 50% CH_3CN in 0.1M AMF water at a flow rate of 5 mL/min. The peaks of desired products are outlined via red squares.

Manual radiosynthesis of [^{18}F]JNJ-46356479 from boronic acid 5

Same procedure was used as those of manual radiolabeling from compound **4**, except the precursor was changed to **5**. The reaction conditions were optimized as shown in Table 1 entries 15 & 16 as well as Table S2. The reaction was monitored by radioTLC and analytical radioHPLC under the same conditions of compound **4**.

Automated Radiosynthesis of [^{18}F]FITM

Similar procedure was used as that for [^{18}F]JNJ-46356479, but the alumina N cartridge was not employed due to the reduced yields. The RCC of [^{18}F]FITM was determined by radioHPLC analysis with a gradient elution (Figure S5, mobile phase: MeCN/0.1%TFA in water (v/v), 1 mL/min, Waters Xbridge BEH C18, 3.5 µm, 4.6 x 150 mm). Gradient: 0-3 min (5% MeCN with 0.1% TFA) isocratic; 3-13 min (5% MeCN with 0.1% TFA to 90% MeCN with 0.1% TFA) linear increase; 13-16 min (90% MeCN with 0.1% TFA) isocratic; 16-17 min (90% MeCN with 0.1% TFA to 5% MeCN with 0.1% TFA) linear decrease; 17-20 min (5% MeCN with 0.1% TFA) isocratic.

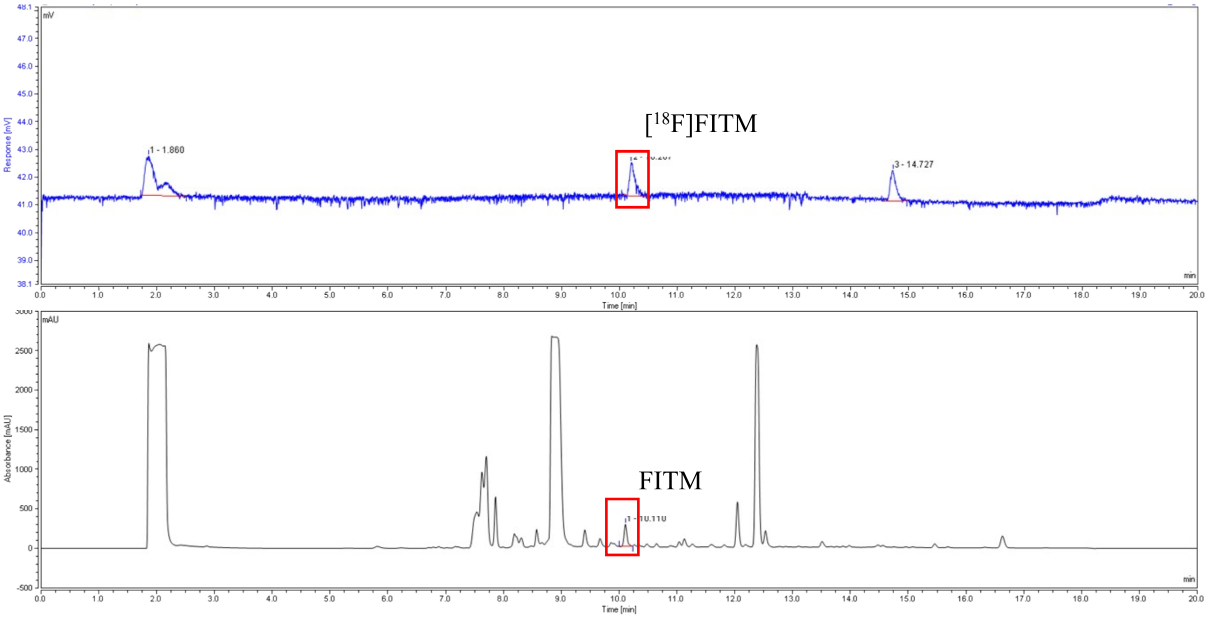

Figure S5. Analytical radioHPLC analysis of [^{18}F]FITM. The radioHPLC trace is shown in blue (top) and the UV trace is shown in black (bottom) under a wavelength of 254 nM.

Optimization of the radiosynthesis of [^{18}F]JNJ-46356479

The radiochemical yields (RCCs) in Tables 1 and 2 were mostly determined by either radioTLC. In practice, the radioTLC EMD TLC Silica gel 60 plates (10 x 2 cm) were spotted with an aliquot (1-5 μL) of crude reaction mixture approximately 1.5 cm from the bottom of the plate (baseline). TLC plates were developed in a chamber containing ethyl acetate (EtOAc) until within 2 cm of the top of the plate (front). Analysis was performed using a Bioscan AR-2000 radio-TLC imaging scanner and WinScan software. A representative RadioTLC chromatogram is shown in Figure S6 for the radiofluorination of [^{18}F]JNJ-46356479 using compound **4**.

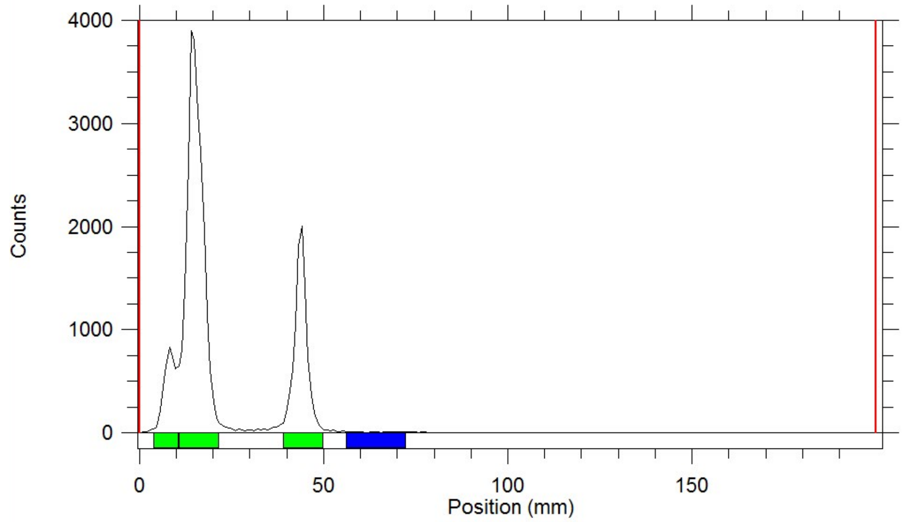

Figure S6. RadioTLC chromatograph of [^{18}F]JNJ-46356479

Reaction condition optimization for [^{18}F]JNJ-46356479 with ester 4

The reaction conditions were optimized below:

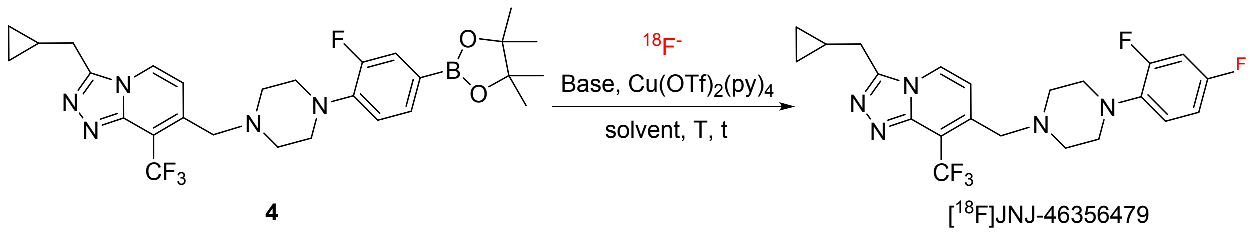

Entry	**4** (mg/equiv.)	**TEAB** (mg/equiv.)	**$Cu(OTf)_2(py)_4$** (mg/equiv.)	**Solvent (mL)**	**T (°C)**	**t (min)**	**RCC by rTLC (%)**
**1**	3.0/1.0	2.7/2.6	18.0/4.9	DMA/*n*BuOH (0.8/0.4)	110	20	22 ± 2 (n = 2)
**2**	3.0/1.0	2.7/2.6	18.0/4.9	DMA/*n*BuOH (0.8/0.4)	130	20	25 ± 2 (n = 5)
**3**	3.0/1.0	2.7/2.6	18.0/4.9	DMA/*n*BuOH (0.8/0.4)	150	20	20 ± 2 (n =2)
**4**	3.0/1.0	8.2/7.8	18.0/4.9	DMA/*n*BuOH (0.8/0.4)	130	20	7 ± 2 (n = 2)
**5**	0.5/1.0	2.7/15.6	18.0/29.4	DMA/*n*BuOH (0.8/0.4)	130	20	2 ± 1 (n = 2)
**6**	1.0/1.0	2.7/7.8	18.0/14.7	DMA/*n*BuOH (0.8/0.4)	130	20	5 ± 1 (n = 2)
**7**	1.5/1.0	2.7/5.2	18.0/9.8	DMA/*n*BuOH (0.8/0.4)	130	20	12 ± 1 (n = 2)
**8**	3.5/1.0	2.7/2.3	18.0/4.3	DMA/*n*BuOH (0.8/0.4)	130	5	28 ± 3 (n = 2)
**9**	2.0/1.0	2.7/3.9	18.0/7.4	DMA/*n*BuOH (0.8/0.4)	130	5	15 ± 1 (n = 2)
**10**	2.0/1.0	2.7/3.9	18.0/7.4	DMA/*n*BuOH (0.8/0.4)	130	10	14 ± 2 (n = 2)
**11**	2.0/1.0	2.7/3.9	18.0/7.4	DMA/*n*BuOH (0.8/0.4)	130	20	15 ± 1 (n = 2)
**12**	2.0/1.0	2.7/3.9	4.5/1.8	DMA/*n*BuOH (0.8/0.4)	130	20	6 ± 1 (n = 2)
**13**	2.0/1.0	2.7/3.9	9.0/3.6	DMA/*n*BuOH (0.8/0.4)	130	20	21 ± 1 (n = 2)
**14**	3.5/1.0	2.7/2.3	9.0/2.1	DMA/*n*BuOH (0.8/0.4)	130	10	28 ± 2 (n = 4)

Table S1. Synthesis and optimization of [^{18}F]JNJ-46356479 via boronic ester precursor **4**

Reaction condition optimization for [^{18}F]JNJ-46356479 with acid 5

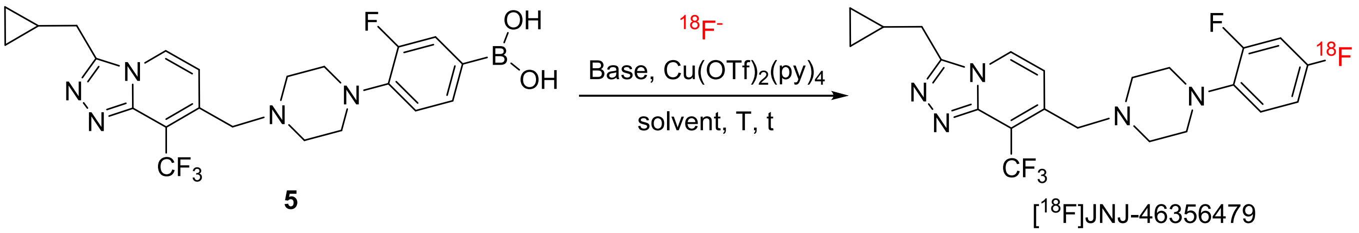

#	5 (mg/equiv.)	TEAB (mg/equiv.)	$Cu(OTf)_2(py)_4$ (mg/equiv.)	Solvent (mL)	T (°C)	t (min)	RCC by rTLC (%)
**1**	2.0/1.0	2.7/3.4	18.0/6.3	DMA/MeOH (0.8/0.4)	110	10	0 (n = 2)
**2**	2.0/1.0	2.7/3.4	18.0/6.3	DMA/MeOH (0.8/0.4)	150	10	0 (n = 2)
**3**	2.0/1.0	2.7/3.4	18.0/6.3	DMA/nBuOH (0.8/0.4)	110	20	5 ± 1 (n = 2)
**4**	2.0/1.0	2.7/3.4	18.0/6.3	DMA/*n*BuOH (0.8/0.4)	130	20	7 ± 2 (n = 2)
**5**	2.0/1.0	2.7/3.4	18.0/6.3	DMA/*n*BuOH (0.8/0.4)	150	20	9 ± 1 (n = 2)
**6**	5.0/1.0	2.7/1.3	18.0/2.5	DMA/*n*BuOH (0.8/0.4)	150	20	13 ± 2 (n = 3)

Table S2. Synthesis and optimization of [^{18}F]JNJ-46356470 via boronic acid precursor **5**

REFERENCES

1. Cid, J. M.; Tresadern, G.; Vega, J. A.; de Lucas, A. I.; del Cerro, A.; Matesanz, E.; Linares, M. L.; Garcia, A.; Iturrino, L.; Perez-Benito, L.; Macdonald, G. J.; Oehlrich, D.; Lavreysen, H.; Peeters, L.; Ceusters, M.; Ahnaou, A.; Drinkenburg, W.; Mackie, C.; Somers, M.; Trabanco, A. A., Discovery of 8-Trifluoromethyl-3-cyclopropylmethyl-7-[(4-(2,4-difluorophenyl)-1-piperazinyl)methyl]-1,2,4-triazolo[4,3-a]pyridine (JNJ-46356479), a Selective and Orally Bioavailable mGlu2 Receptor Positive Allosteric Modulator (PAM). *Journal of Medicinal Chemistry* **2016,** *59* (18), 8495-8507.

2. Taylor, N. J.; Emer, E.; Preshlock, S.; Schedler, M.; Tredwell, M.; Verhoog, S.; Mercier, J.; Genicot, C.; Gouverneur, V., Derisking the Cu-Mediated F-18-Fluorination of Heterocyclic Positron Emission Tomography Radioligands. *Journal of the American Chemical Society* **2017,** *139* (24), 8267-8276.

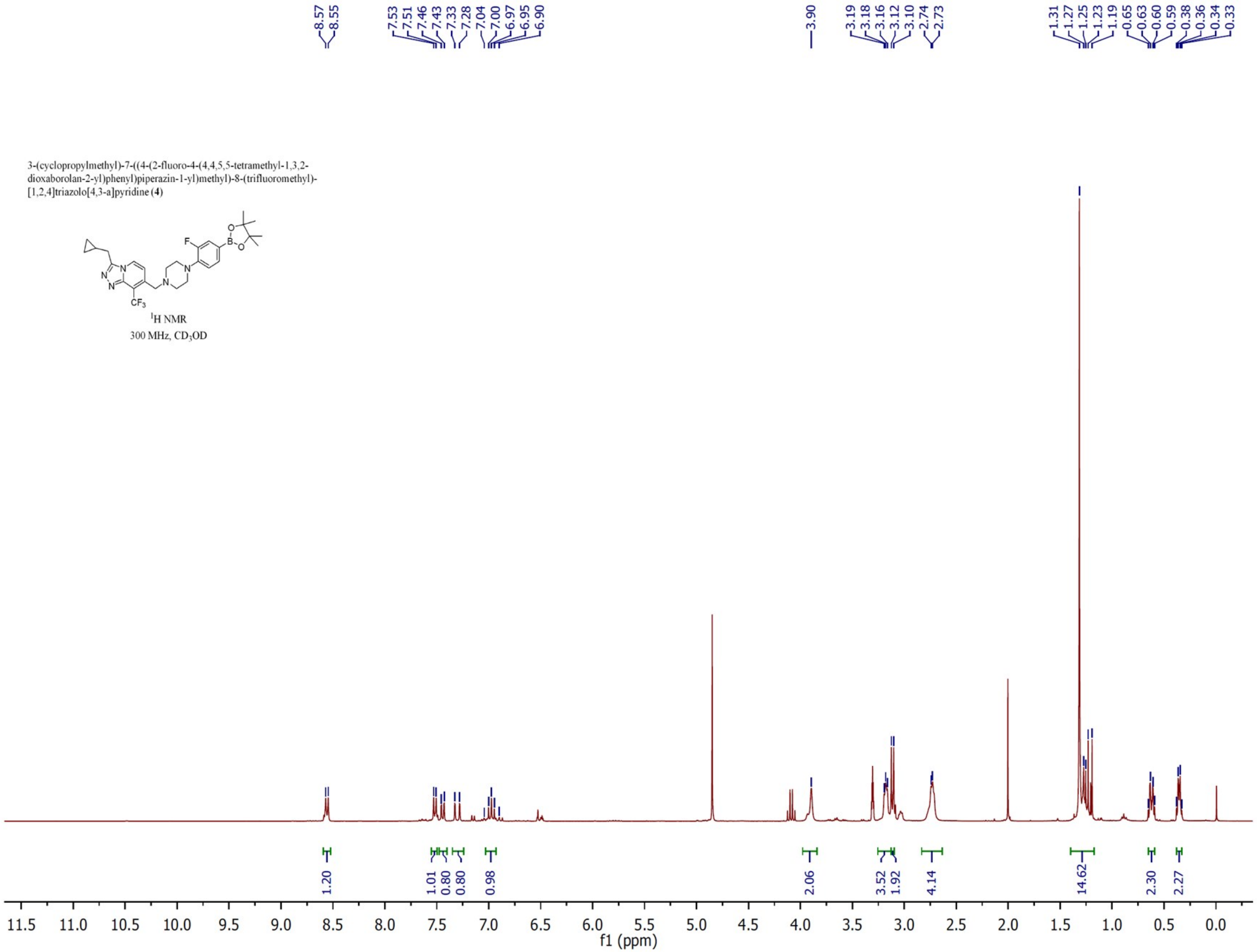

3-(cyclopropylmethyl)-7-((4-(2-fluoro-4-(4,4,5,5-tetramethyl-1,3,2-dioxaborolan-2-yl)phenyl)piperazin-1-yl)methyl)-8-(trifluoromethyl)-[1,2,4]triazolo[4,3-a]pyridine (**4**)

^{1}H NMR

300 MHz, CD_3OD

3-(cyclopropylmethyl)-7-((4-(2-fluoro-4-(4,4,5,5-tetramethyl-1,3,2-dioxaborolan-2-yl)phenyl)piperazin-1-yl)methyl)-8-(trifluoromethyl)-[1,2,4]triazolo[4,3-a]pyridine (**4**)

^{13}C NMR

125 MHz, CD_3OD

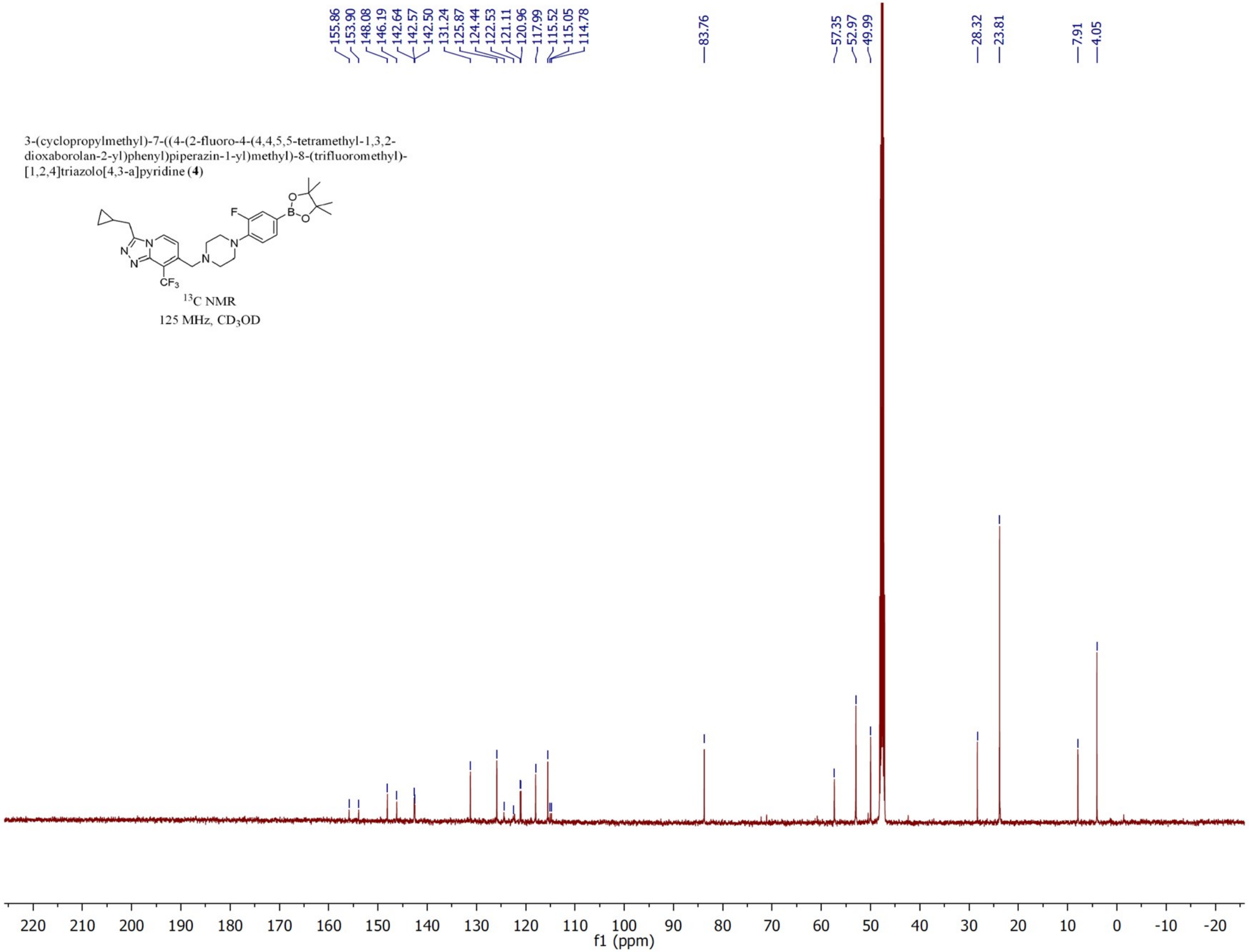

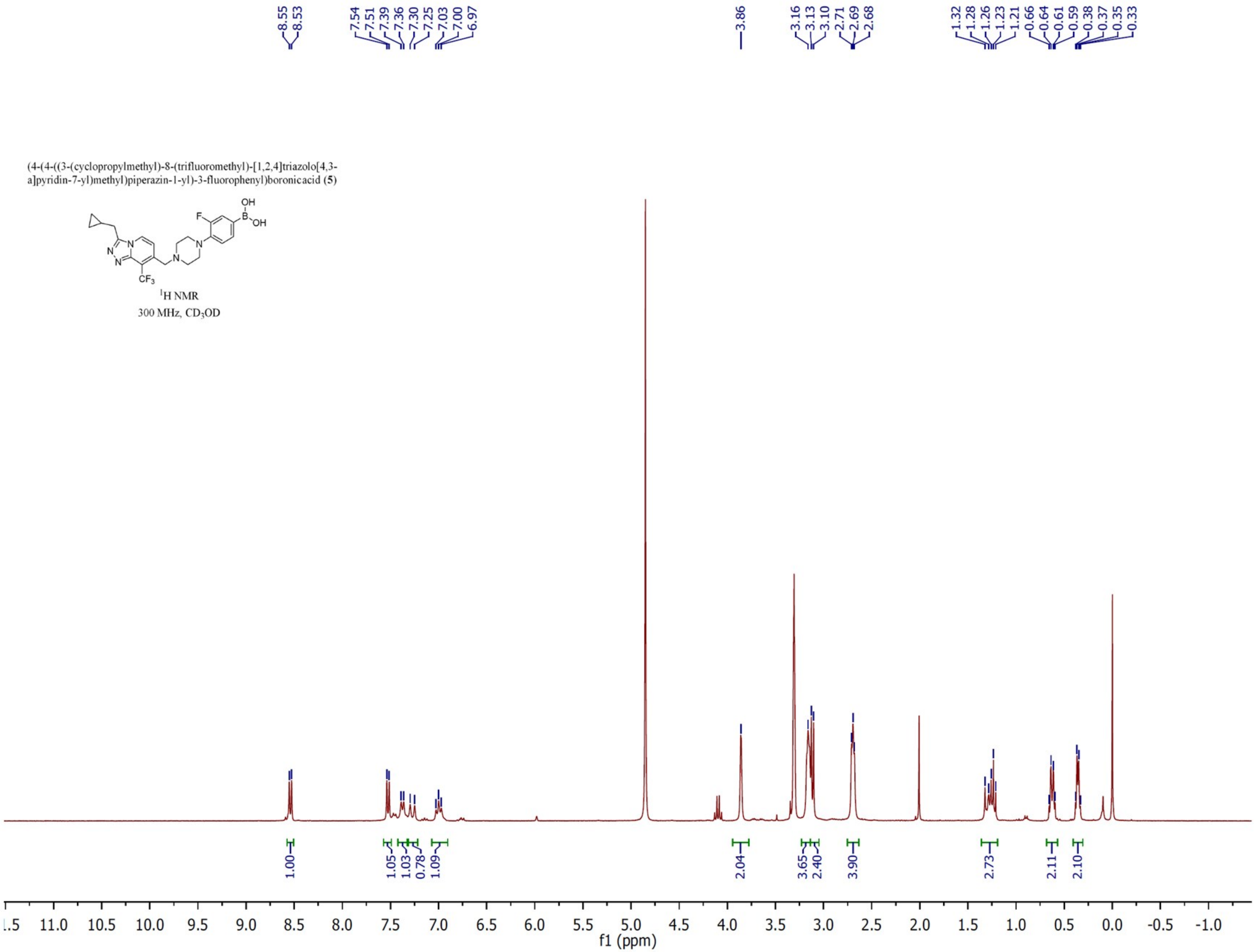
8.55
8.53
7.54
7.51
7.39
7.36
7.30
7.25
7.03
7.00
6.97
3.86
3.16
3.13
3.10
2.71
2.69
2.68
1.32
1.28
1.26
1.23
1.21
0.66
0.64
0.61
0.59
0.38
0.37
0.35
0.33
(4-(4-((3-(cyclopropylmethyl)-8-(trifluoromethyl)-[1,2,4]triazolo[4,3-a]pyridin-7-yl)methyl)piperazin-1-yl)-3-fluorophenyl)boronicacid (5)
OH
F
B
OH
N
N
N
N
N
CF3
1H NMR
300 MHz, CD3OD
1.00
1.05
1.03
0.78
1.09
2.04
3.65
2.40
3.90
2.73
2.11
2.10
11.0
10.5
10.0
9.5
9.0
8.5
8.0
7.5
7.0
6.5
6.0
5.5
5.0
4.5
4.0
3.5
3.0
2.5
2.0
1.5
1.0
0.5
0.0
-0.5
-1.0
f1 (ppm)

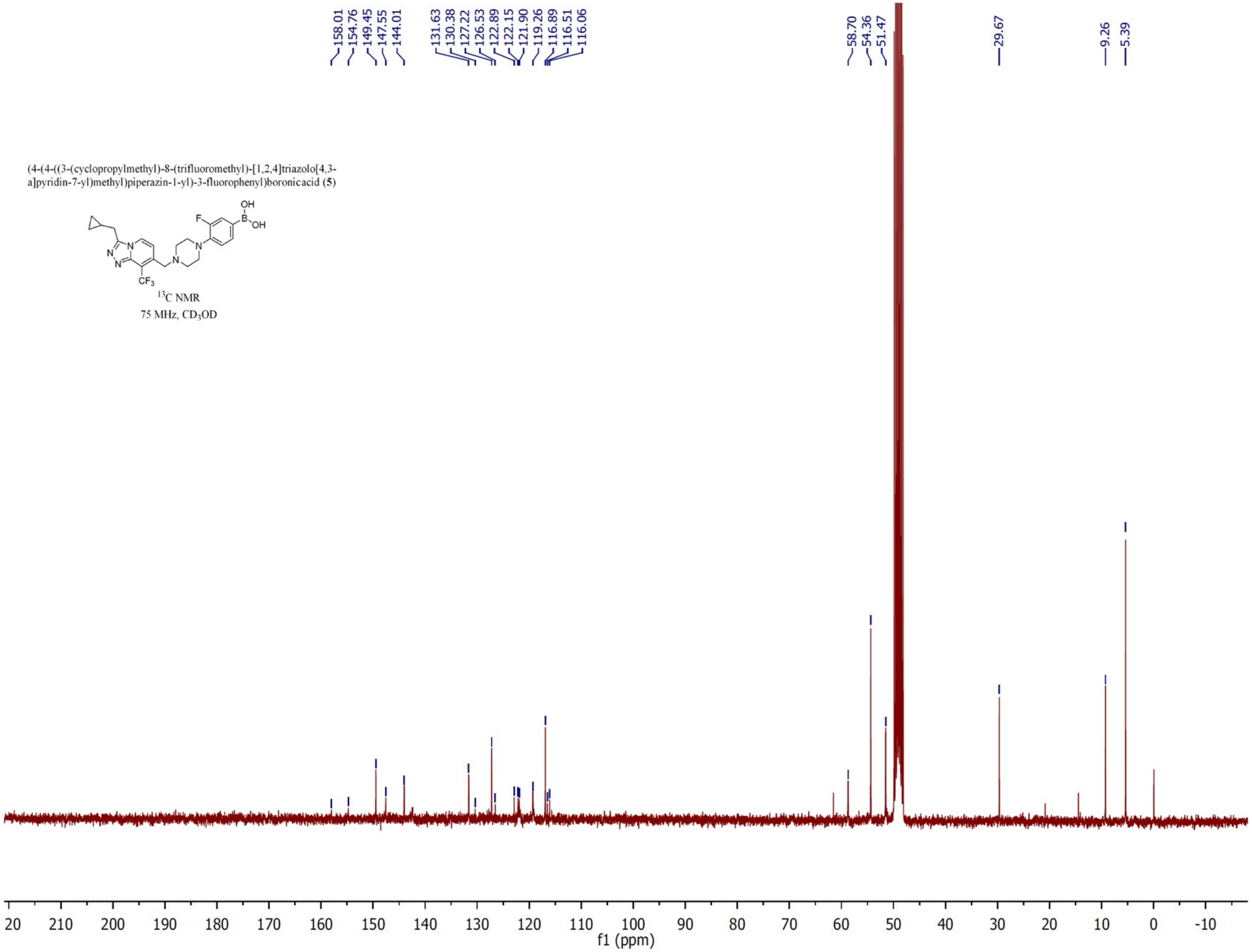
(4-(4-((3-(cyclopropylmethyl)-8-(trifluoromethyl)-[1,2,4]triazolo[4,3-a]pyridin-7-yl)methyl)piperazin-1-yl)-3-fluorophenyl)boronicacid (5)
^{13}C NMR
75 MHz, CD_3OD
158.01
154.76
149.45
147.55
144.01
131.63
130.38
127.22
126.53
122.89
122.15
121.90
119.26
116.89
116.51
116.06
58.70
54.36
51.47
29.67
9.26
5.39
f1 (ppm)
